# Differences in complications between colonoscopy and esophagogastroduodenoscopy in Japan using large-scale health insurance claims data

**DOI:** 10.1055/a-2689-6049

**Published:** 2025-09-12

**Authors:** Naohisa Yoshida, Hideki Ishikawa, Michihro Mutoh, Naoto Iwai, Reo Kobayashi, Ken Inoue, Ryohei Hirose, Osamu Dohi, Yoshito Itoh, Azusa Yoda, Ayako Maeda-Minami, Yasunari Mano

**Affiliations:** 1 12898 Department of Molecular Gastroenterology and Hepatology, Graduate School of Medical Science, Kyoto Prefectural University of Medicine, Kyoto, Japan; 2 Department of Molecular-Targeting Prevention, Graduate School of Medical Science, Kyoto Prefectual University of Medicine, Osaka, Japan; 3 Department of Infectious Diseases, Graduate School of Medical Science, Kyoto Prefectural University of Medicine, Kyoto, Japan; 4 26413 Department of Clinical Drug Informatics, Faculty of Pharmaceutical Sciences, Tokyo University of Science, Tokyo, Japan

**Keywords:** Endoscopy Lower GI Tract, CRC screening, Quality and logistical aspects, Performance and complications

## Abstract

**Background and study aims:**

Analyses of colonoscopy (CS) and esophagogastroduodenoscopy (EGD) complications is crucial for further promoting use of endoscopy. This study analyzed rates of severe complications of CS compared with those of EGD using big data.

**Patients and methods:**

As a study population, we retrospectively used commercially anonymized health insurance claims data covering 3,050,954 patients from January 2010 to December 2020. Patients ≥ 50 years old who underwent CS or EGD without treatment were included in the study. The main outcomes were differences in rates of hemorrhage, perforation, and fatal events between EGD and CS, and risk factors of each complication comparing CS with EGD.

**Results:**

Among 290,470 CSs (male: 182,910, female: 107,560, median age [range]: 58 [50–75]) and 726,075 EGDs (male: 412,365, female: 313,710, 58 [50–75]), rates of hemorrhage, perforation, and fatal events for EGD and CS were 0.0069% vs. 0.0069% ( *P* = 0.558), 0.0006% vs. 0.0024% ( *P* = 0.008), and 0.00028% vs. 0.00034% ( *P* = 0.648), respectively. Rates of hemorrhage for cases aged 50 to 64 and 65 to 75 years were 0.0059% vs. 0.0110% ( *P* = 0.042) for EGD and 0.0061% vs. 0.0108% for CS ( *P* = 0.264). Risks of hemorrhage for comparing CS to EGD were significant for biopsy (adjusted odds ratio [aOR] 95% confidence interval [CI] 2.75 [1.15–6.21]; *P* = 0.017) and antithrombotics (aOR 12.48; 95% CI 1.80–247.14; *P* = 0.026). Those for perforation were significant for ages 50 to 64 years (aOR 9.58; 95% CI 2.17–66.10; *P* = 0.006) and male sex (11.76 [1.85–222.65], *P* = 0.025).

**Conclusions:**

Compared with EGD, CS had a higher rate of perforation but not hemorrhage. Complication rates in CS did not differ by age.

## Introduction

Globally in terms of cancer incidence in 2022, colorectal cancer (CRC) ranks third with 1,926,425 cases 1 . It is rapidly increasing because there were 1,096,601 CRC cases in 2018. In 2021, incidence of CRC in Japan was 154,585 people per year, the highest among all cancers 2 . CRC is also increasing because the number of cases in 2012 was 134,575.

Fecal occult blood tests (FOBTs) have been performed for CRC screening in many countries, including Japan. Many randomized controlled trials and case-control studies conducted in Europe, the United States, and Japan have reported definite effects of FOBT in reducing CRC deaths 3 4 5 6 7 . However, there are several issues, such as a low participation rate for FOBT, low positive predictive value, and low rate of receiving colonoscopy (CS) despite a positive result 8 9 10 11 .

Efficacy of CS in reducing morbidity and mortality of CRC has been reported in previous reports 12 13 14 15 . In fact, screening CS for people aged ≥ 50 years in the United States has helped reduce the number of CRC deaths 16 . However, only a few countries, such as the United States, Germany, and Poland, have adopted CS for cancer screening. Safety of CS is important as CRC screening. In Japan, a nationwide observational study examining complications of endoscopy for 1 week in each center from 2019 to 2021 showed that the number of hemorrhages, perforations, and fatal events with CS for both asymptomatic and symptomatic reasons was 0.0081% (5 cases), 0.0081% (5 cases), and 0% (0 cases), respectively, among 61,083 cases 17 . On the other hand, esophagogastroduodenoscopy (EGD) is performed for upper gastrointestinal symptoms and has been adopted as gastric cancer screening in Japan and Korea 18 . In the Japanese study, EGD was more frequent than CS and hemorrhage, perforation, and fatal event rates for screening and symptomatic EGD were 0.0031%, 0.0007%, and 0%, respectively, among 126,304 cases 17 . However, few studies on CS and EGD have examined the current frequency of complications by sex and age and investigated their specific risk factors. Given the high global prevalence of CRC and gastric cancer, a detailed analysis of CS and EGD complications is crucial for further promoting use of endoscopy. In this study, we analyzed severe complications of CS and EGD, identified their associated risk factors, and compared these complications between CS and EGD using large-scale health insurance claims data.

## Patients and methods

### Study population

We used commercial anonymized information from 3,050,954 patients aged ≥ 50 years in the JMDC database (JMDC Inc., Tokyo, Japan; https://www.jmdc.co.jp/en/index ) from January 2010 to December 2020, in which society-managed health insurance claims data from workers and their families in large companies were registered. This database consists of statements about medical expenses of hospitalization, outpatient, dispensing, and health checkup data and contains demographic characteristics (e.g. age and sex), procedures (blood examinations, endoscopy, surgery, etc.) including their date, diagnoses of disease coded, and prescribed drugs from outpatient and hospitalization care. If multiple medical institutions were used, each individual’s data were tracked in chronological order.

**Fig. 1:**
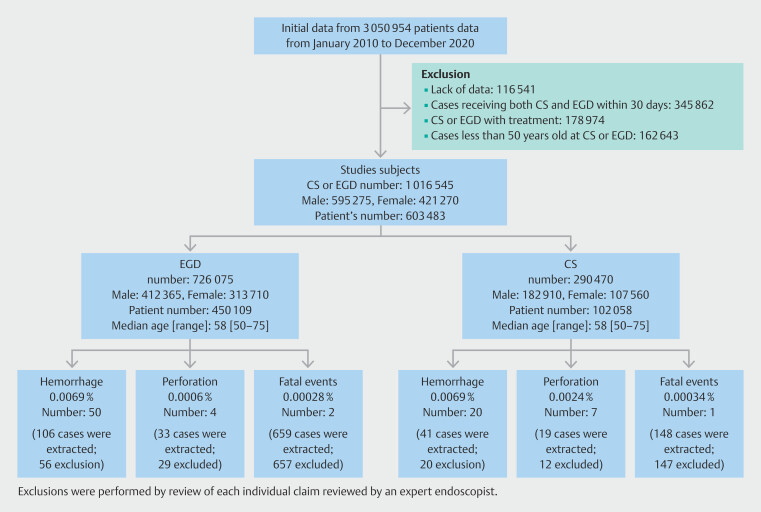
Study flow.

We extracted data fom 1,820,565 patients with CS or EGD with and without treatment, using the Japanese standard code for medical procedures related to them (Supplementary Table 1), referring to a previous paper 19 . CS or EGD without treatment was both symptomatic and for screening, including diagnostic purposes, and included a biopsy. CS and EGD with lesion resection included polypectomy, endoscopic mucosal resection, and endoscopic submucosal dissection. After excluding 116,541 cases without appropriate data, 345,862 cases receiving both CS and EGD performed within 30 days, 178,974 cases of CS or EGD with treatment, and 162,643 cases in patients younger than age 50 years at CS or EGD, we finally analyzed 1,016,545 cases, including 290,470 CSs and 726,075 EGDs ( Fig. 1 ). Using these data, we obtained information on comorbidities, medical procedures, and drugs from both inpatients and outpatients.

### Definitions of complications

**Fig. 2:**
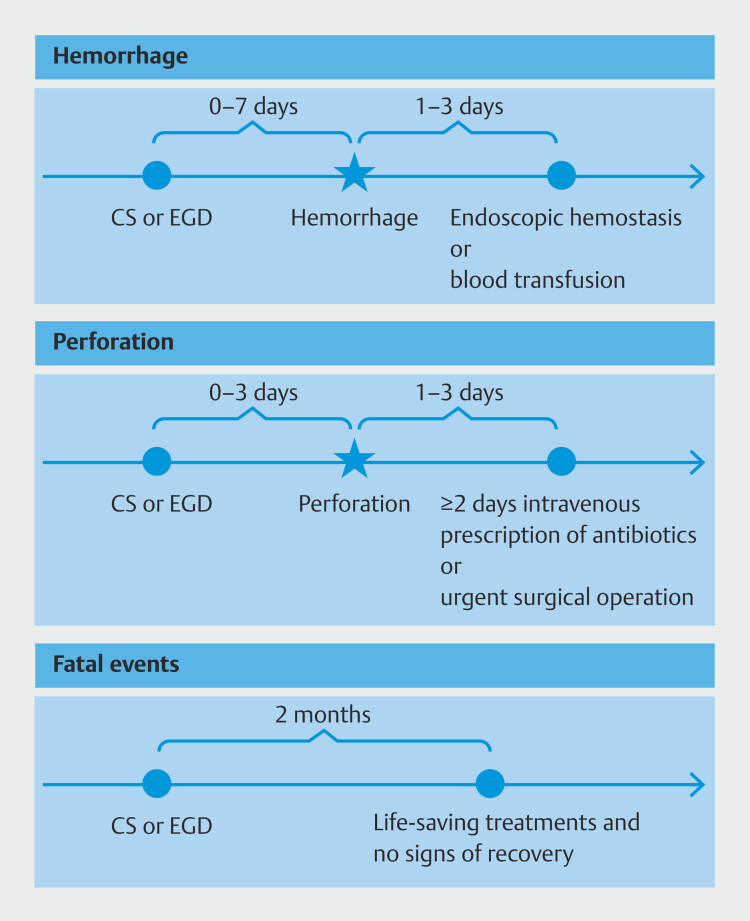
Definitions of hemorrhage and perforation using the information from health insurance claim data.

Definitions of three major complications of hemorrhage, perforation, and fatal events using health insurance claims data were arranged for this study, following the approach of our previous paper 19 . Data on CS and EGD performed 0 to 7 days before the onset date of hemorrhage were extracted ( Fig. 2 ). We analyzed severe cases that required either endoscopic hemostasis or blood transfusion within 3 days of the onset date, using Japanese standard disease codes and medical procedures (Supplementary Table 2). We also included cases of hemorrhage due to a biopsy that required endoscopic hemostasis or blood transfusion. Data on CS and EGD performed 0 to 3 days before the onset date of perforation were extracted ( Fig. 2 ). We analyzed severe cases that needed treatment, such as those with ≥ 2 days of intravenous (IV) antibiotics or urgent surgery, using Japanese standard disease codes and medical procedures within 3 days after onset of perforation (Supplementary Table 3 and Supplementary Table 4) 19 .

For fatal events, given that the JMDC database lacked information on the date of death, we estimated fatal events as patients who withdrew from the same health insurance program within 2 months after the last EGD or CS procedure and received various life-saving treatments, such as endotracheal intubation, heart massage, electric counter shock, cardiac intervention for cardiac infarction, cerebral-infarction treatments, and various urgent cardio-cerebral drugs, including adrenalin, dopamine, dobutamine, edaravone, and alteplase, just after EGD or CS and showed no signs of recovery using Japanese standard disease codes and medical procedures according to our previous paper (Supplementary Table 5) 19 . The JMDC database contains monthly medical claims and claims data are censored because of withdrawal from the health insurance union due to death. If a patient dies at the end of the month, withdrawal from the health insurance union may occur in the following month. Thus, we defined 2 months as the duration of fatal events.

From the extracted cases of hemorrhage, perforation, and fatal events, an expert endoscopist (N.Y.) excluded cases with diseases not related to CS or EGD, thoroughly checking individual claims data 2 to 3 months before and after the events and the target CS or EGD. We conducted intentional overestimation when estimation of complications was difficult owing to a lack of information. In addition, when any colorectal or gastric surgeries were performed before or after CS or EGD among these extracted cases, the adverse events that were estimated to be related to the colorectal or gastric surgeries were excluded.

### Evaluation items

Evaluation items in this study were differences in rates of hemorrhage, perforation, and fatal events between CS and EGD. Rates according to age (< 65 vs. ≥ 65 years old) and sex were also examined. Risk of hemorrhage and perforation (odds ratio [OR] and 95% confidence interval [CI]) when comparing CS with EGD was examined using logistic regression analyses. We also analyzed various risk factors for hemorrhage and perforation using univariate and multivariate logistic regression analyses. Risk factors analyzed were age (< 65 and ≥ 65 years), sex, and comorbidities (e.g. heart disease, peripheral vessel disease, cerebrovascular disease, diabetes mellitus, liver disease, renal disease, hypertension, cancer, ulcerative colitis, biopsies, hemodialysis, and antithrombotic drugs) (Supplementary Table 6).

This study was conducted in accordance with the World Medical Association Declaration of Helsinki and approved by the ethics committee of Tokyo University of Science (23039).

### Statistical analyses

All statistical analyses were performed using SAS software program (version 9.4; SAS Institute, Cary, North Carolina, United States). Although some patients who contributed data to this study had more than one CS or EGD procedure, the quantities observed in different CS or EGD were assumed to be independent observations for purposes of data analysis. The chi-squared test with Yates correction for categorical variables and Mann–Whitney U test for continuous variables were used. Risk factors for hemorrhage and perforation were analyzed using a logistic regression analysis model. Univariate logistic regression analysis was performed to screen variables associated with presence or absence of complications, such as hemorrhage and perforation, in colonoscopy with and without lesion resection. Variables such as Charlson Comobidity Index, biosp, antithrombotic drug, sex, and age were entered into multivariate analysis to identify any independent risk factors associated with complications after adjusting for contributions of other variables. ORs and 95% CIs were estimated in univariate and multivariate logistic analyses. Statistical significance was set at *P* < 0.05.

## Results

In the EGD and CS groups, there were 726,075 and 290,470 patients, respectively ( Table 1 ). There was a significant age difference in the two groups (median [interquartile]) (58 [54–63] vs. 58 [53–62], *P* < 0.001) due to the huge number of patients and different age distribution. In addition, there were significant differences in sex, comorbidities, procedures, and medications between the two groups.

**Table:** **Table 1** Case characteristics.

	EGD	CS	*P* value
Case number	726,075	290,470	
Age (years; median [IQR] (range])	58 [54–63] (50–75)	58 [53-62] (50–75]	< 0.001
50–54, n (%)	204,006 (28.10)	92,289 (31.77)	
55–59, n (%)	207,641 (28.60)	84,934 (29.24)	
60–64, n (%)	178,078 (24.53)	67,125 (23.11)	
65–69, n (%)	89,163 (12.28)	30,987 (10.67)	
≥ 70, n (%)	47,187 (6.50)	15,135 (5.21)	
Sex, n (%)			
Male	412,365 (56.79)	182,910 (62.97)	< 0.001
Female	313,710 (43.21)	107,560 (37.03)	
Comorbidities, n (%)			
Heart disease	54,911 (7.56)	19,234 (6.62)	< 0.001
Cerebrovascular disease	34,126 (4.70)	11,452 (3.94)	< 0.001
Liver disease	117,245 (16.15)	37,990 (13.08)	< 0.001
Diabetes mellitus	88,974 (12.25)	21,931 (7.55)	< 0.001
Renal disease	17,103 (2.36)	4,988 (1.72)	< 0.001
Hypertension	202,530 (27.89)	68,186 (23.47)	< 0.001
Cancer	81,521 (11.23)	31,964 (11.00)	0.001
Crohn’s disease	662 (0.09)	1,474 (0.51)	< 0.001
Ulcerative colitis	3,066 (0.42)	1,7192 (5.92)	< 0.001
CCI (median [IQR] (range))	0 [0–2] (0–18)	0 [0–2] [0–17]	< 0.001
Procedures, n (%)			
Biopsy	232,199 (31.98)	56,999 (19.62)	< 0.001
Hemodialysis	2,103 (0.29)	494 (0.17)	< 0.001
Medications n (%)			
Antithrombotic drug	35,237 (4.85)	13,490 (4.64)	< 0.001
Warfarin	3,088 (0.43)	1,440 (0.50)	< 0.001
DOAC	4,579 (0.63)	2,488 (0.86)	< 0.001
Antiplatelet	28,821 (3.97)	10,222 (3.52)	< 0.001
CCI, Charlson comorbidity index; CS, colonoscopy; DOAC, direct oral anticoagulant; EGD, esophagogastroduodenoscipy; DOAC, direct oral anticoagulant; IQR, interquartile range.			

Rates of hemorrhage, perforation, and fatal events for the EGD and CS groups were 0.0069% vs. 0.0069% ( *P* = 0.558), 0.0006% vs. 0.0024% ( *P* = 0.008), and 0.00028% vs. 0.00034% ( *P* = 0.648), respectively ( Table 2 ) ( Fig. 1 ). There was a significant difference in rates of hemorrhage for patients aged 50 to 64 and 65 to 75 years in the EGD group (0.0059% vs. 0.0110%, *P* = 0.042). There was no significant difference for those in the CS group (0.0061% vs. 0.0108%, *P* = 0.264). In addition, there were no significant differences in perforation or fatal events according to age and sex for the EGD and CS groups.

**Table:** **Table 2** Rates of hemorrhage, perforation, and fatal events for EGD and CS according to age and sex.

			Overall number (n)	Hemorrhage (n)	Rate (%)	95% CI	*P* value	Perforation (n)	Rate (%)	95% CI	*P* value	Fatal events (n)	Rate (%)	95% CI	*P* value
EGD	Overall		726,075	50	0.0069	(0.0067–0.0071)		4	0.0006	(0.0005–0.0006)		2	0.0003	(0.0002–0.0003)	
	Age (years)														
		< 65	589,725	35	0.0059	(0.0042–0.0083)	0.042	2	0.0003	(0.0000–0.0013)	0.337	2	0.0003	(0.0000–0.0013)	0.821
		≥ 65	136,350	15	0.0110	(0.0065–0.0183)		2	0.0014	(0.0000–0.0057)		0	0	–	
	Sex														
		Male	412,365	30	0.0073	(0.0070–0.0075)	0.647	1	0.0002	(0.0002–0.0003)	0.435	1	0.0002	(0.0002–0.0003)	0.603
		Female	313,710	20	0.0064	(0.0061–0.0067)		3	0.0010	(0.0009–0.0011)		1	0.0003	(0.0003–0.0004)	
CS	Overall		290,470	20	0.0069	(0.0066–0.0072)		7	0.0024	(0.0022–0.0026)		1	0.0003	(0.0003–0.0004)	
	Age (years)														
		< 65	244,348	15	0.0061	(0.0036–0.0102)	0.264	6	0.0025	(0.0000–0.0055)	0.687	1	0.0004	(0.0000–0.0026)	0.35
		≥ 65	46,122	5	0.0108	(0.0038–0.0262)		1	0.0022	(0.0000–0.0136)		0	0	–	
	Sex														
		Male	182,910	15	0.0082	(0.0078–0.0086)	0.265	5	0.0027	(0.0025–0.0030)	0.942	0	0	–	0.788
		Female	107,560	5	0.0047	(0.0042–0.0051)		2	0.0019	(0.0016–0.0021)		1	0.0009	(0.0008–0.0011)	
CS, colonoscopy; EGD, esophagogastroduodenoscopy.															

Logistic regression analysis was used to compare overall risks of hemorrhage and perforation between the EGD and CS groups ( Table 3 ). Adjusted ORs (95% CIs) comparing the CS group with the EGD group were 1.17 (0.68–1.94) ( *P* = 0.558) for hemorrhage and 5.46 (1.62–21.17) ( *P* = 0.008) for perforation.

**Table:** **Table 3** Comparison of hemorrhage and perforation between EGD and CS using logistic regression analysis.

		Events	Rate (%)	Crude OR (95% CI)	*P* value	Adjusted OR (95% CI)	*P* value
Hemorrhage	EGD (N=726,075)	50	0.0069	N/A	–	N/A	–
	CS (N=290,470)	20	0.0069	1.00 (0.58–1.65)	1.000	1.17 (0.68–1.94)	0.558
Perforation	EGD (N=726,075)	4	0.0006	N/A	–	N/A	–
	CS (N=290,470)	7	0.0024	4.37 (1.32–16.7)	0.019	5.46 (1.62–21.17)	0.008
Adjusted by age, sex, biopsy, Charlson Comorbidity Index, antithrombotic drugs. CS, colonoscopy; C, confidence interval; EGD, esophagogastroduodenoscopy; N/A, not applicable; NC, not calculated; OR, odds ratio.							

Risk factors for hemorrhage comparing the EGD groups to the CS groups were examined ( Table 4 ). There were no significant differences in age or sex between the EGD and the CS groups. In contrast, there were significant differences in biopsy ( *P* = 0.033) and antithrombotic drugs ( *P* = 0.026) between the groups and the adjusted ORs (95% CIs) were 2.75 (1.15–6.20) and 12.48 (1.80–247.14). In addition, the history of any type of cancer was significant (interaction *P* value, *P* = 0.031).

**Table:** **Table 4** Risk of hemorrhage between EGD and CS according to various factors.

	EGD	CS	Adjusted OR (95% CI)*	*P* value	Interaction *P* value
Age (years)					
≥ 65	15/136,350	5/46,122	1.31 (0.42-3.41)	0.606	0.911
< 65	35/589,725	15/244,348	1.11 (0.58-2.00)	0.747	
Sex					
Male	30/412,365	15/182,910	1.26 (0.66-2.32)	0.470	0.377
Female	20/313,710	5/107,560	0.88 (0.29-2.19)	0.801	
CCI					
≥ 1	33/271,251	9/91,517	0.83 (0.37-1.67)	0.619	NC
0	17/454,824	11/198,953	NC		
Biopsy					
+	15/232,199	9/56,999	2.75 (1.15-6.20)	0.017*	0.033*
–	35/493,876	11/233,471	0.77(0.37-1.48)	0.455	
Antithrombotic drug					
+	1/35,237	4/13,490	12.48 (1.80-247.14)	0.025*	0.026*
–	49/690,838	16/276,980	0.95 (0.52-1.64)	0.861	
*Adjusted by age, sex, biopsy, Charlson Comorbidity Index, antithrombotic drugs. CI, confidence interval; CS, colonoscopy; EGD, esophagogastroduodenoscopy; NC, not calculated; OR, odds ratio.					

Risk of perforation according to age and sex between EGD and CS was also examined ( Table 5 ). There were significant differences in proportions of age < 65 years ( *P* = 0.006) and male sex ( *P* = 0.026) between the groups. Adjusted ORs (95% CIs) were 9.58 (2.17–66.10) and 11.76 (1.85–227.65).

**Table:** **Table 5** Risk of perforation between EGD and CS according to age and sex.

	EGD	CS	Adjusted OR (95%CI)	*P* value	Interaction *P* value
Age (years)					
≥ 65	2/136,350	1/46,122	1.45 (0.07-15.37)	0.762	0.485
< 65	2/589,725	6/244,348	9.58 (2.17-66.10)	0.006	
Sex					
Male	1/412,365	5/182,910	11.76 (1.85-227.65)	0.026	0.216
Female	3/313,710	2/107,560	2.79 (0.36-17.18)	0.267	
CCI					
≥ 1	2/271,251	4/91,517	7.20(1.38-52.64)	0.024	0.699
0	2/454,824	3/198,953	3.85 (0.62-29.86)	0.146	
Biopsy					
+	1/232,199	4/56,999	2.75 (1.15-6.21)	0.017	0.146
–	3/493,876	3/233,471	0.65 (0.32-1.25)	0.221	
Antithrombotic drug					
+	1/35,237	0/13,490	NC		NC
–	3/690,838	7/276,980	0.95 (0.52-1.64)	0.861	
Adjusted by age, sex, biopsy, Charlson Comorbidity Index, antithrombotic drugs. CI, confidence interval;.CS, colonoscopy; EGD, esophagogastroduodenoscopy; N/A, not applicable; NC,not calculated; OR, odds ratio.					

## Discussion

Regarding the rate of complications in CS, a large-scale review of CS without treatment, including 21 studies from Germany, the United States, Canada, the U.K., and Lithuania, showed hemorrhage and perforation rates of 0.026% and 0.005% respectively 20 . Ranges of these rates varied from 0.001% to 0.687% and 0.005% to 0.085%, respectively. Another review from Germany showed that rates of hemorrhage and perforation were 0.001% and 0.012% for CS 21 . These low rates suggest that CS is safe. However, the safety of CS is affected by various factors, including region and endoscopists, and should be examined in each country. In the current study, hemorrhage and perforation rates were 0.0069% and 0.0024%, respectively. These rates are not significantly different from those reported in previous studies 17 20 21 .

In Japan, gastric cancer screening has been performed using gastrography or EGD every 2 years for people ≥50 years old since 2014. In the current study, rates of complications were 0.0069%, 0.0006%, and 0.00028%, respectively. These rates were comparable to those reported in the previous study 17 . This indicates acceptable validity of the analysis in the current study. In addition, differences in rates of hemorrhage and fatal events between the CS and EGD groups were not significant. In contrast, the perforation rate was significantly higher in the CS group than in the EGD group, although both rates were low. For adoption of screening CS, we should take care regarding the risk of perforation.

With respect to analysis of risk factors for complications, a large-scale cohort study of 1,144,900 people from Canada showed that risk factors associated with examinations and treatments were older age, high American Society of Anesthesiologists class, female sex, inpatient status, CS with treatment, polyp size > 10 mm, and surgeon-performed procedure 22 . Regarding age, compared with patients aged < 60 years, ORs for patients aged 60 to 74 and ≥ 75 years were 2.69 (95% CI 1.83–3.98, *P* < 0.001) and 5.63 (95% CI 3.73–8.49, *P* < 0.001), respectively. A cohort study of 947,061 patients from France also showed that the OR (95% CI) for patients aged 60 to 69 years was 2.91 (1.66–5.10), for patients aged 70 to 79 years it was 5.38 (3.08–9.40), and for patients aged ≥ 80 years it was 7.51 (4.20–13.45), compared with patients aged < 60 years 23 . In the same study, risk factors for hemorrhage (OR, 95% CI) were age 60 to 69 years (1.70, 1.18–2.43), age 70 to 79 years (2.55, 1.77–3.66), age ≥ 80 years (3.23, 2.21–4.73), emergency CS, resection of polyps ≥ 1 cm in size, chronic disease, and male sex. Previously, we analyzed complications in 341,852 CSs without treatment using the same health insurance claims data from January 2005 to August 2018 19 . Risk factors for hemorrhage in CS were not identified in multivariate analysis, but male sex and warfarin exposure were identified in univariate analysis. Risk factors for CS perforation were not identified in either univariate or multivariate analyses. In the current study, there was no significant increase in hemorrhage and perforation rates for CS according to age. The significant risk comparing CS with EGD was biopsy and antithrombotic drugs for hemorrhage and age < 65 years and male sex for perforation. To our knowledge, this is the first report to analyze risk factors for complications comparing CS and EGD.

Regarding mortality associated with CS with and without lesion resection, this rate was 2.9 per 100,000 (0.0029%) in a review paper 14 . Another study showed that the mortality rate ranged from 0.007% to 0.07% 24 . However, these rates included all indications for CS. The current study did not show the death rate, but instead showed fatal events for CS without treatment as a proxy for death. In a large-scale report from Japan, which was only written in Japanese, a survey using a questionnaire sent to 544 institutions from 2008 to 2012 showed rates of hemorrhage, perforation, and death in 3,815,118 CSs without treatment to be 0.0019% (n = 75), 0.005% (n = 200), and 0.0004% (n = 17), respectively 25 . Causes of death were embolisms, cardiac arrest, and perforation. The rate of fatal CS events in the current study was 0.00034%, which is comparable to these reports. However, fatal events that were originally defined in the current study were not actual death rates. A validation study of these definitions is expected in the future to compare our complication rates with those of other studies.

Several limitations associated with the present study warrant mention. This was a study using retrospective health insurance claims data; therefore, the study cohort included all patients who underwent CS or EGD regardless of symptoms, FIT results, or personal/family history of CRC. The database was developed by extracting data from several health insurance systems that were used mainly by employees at large companies and their family members. In addition, we extracted cases of hemorrhage, perforation, and fatal events using our original definition based on disease names, medical procedures, and drug information. Hemorrhage without endoscopic hemostasis or blood transfusion (e.g. transcatheter or surgical intervention without blood transfusion), and minor perforations without IV antibiotics were not extracted. A validation study for extracting cases with hemorrhage, perforation, and fatal events was not performed. We conducted intentional overestimation when estimation of complications was difficult due to a lack of information. Regarding CS, we were unaware of individual endoscopist experience, such as whether they were experts or non-experts, and we could not determine patient status as inpatient or outpatient, although it might affect complication rates. We did not analyze complications related to bowel preparation or sedation. Hemorrhage and perforation developing more than 7 or 3 days after CS or EGD were not examined. Our study included only 15,135 patients aged ≥ 70 years (5.2%). We did not conduct a validity study for our analysis; therefore, some cases may not have been appropriately identified in each analysis.

## Conclusions

The CS group did not show a higher rate of hemorrhage compared with the EGD group overall. However, biopsy and use of antithrombotic drugs significantly increased risk of hemorrhage in the CS group. The CS group had a higher rate of perforation than the EGD group. In the CS group, age < 65 years, male sex, Charlson Comorbidity Index ≥ 1, and biopsy were identified as risk factors for perforation. There was no significant difference in rate of fatal events between the two groups.

### Data Availability Statement

The data analyzed in this study are available from JMDC Co., Ltd.. Restrictions apply to the availability of these data, which were used under license for this study. Data are available from the authors upon reasonable request with the permission of JMDC Co., Ltd..

## Supplementary Material

Supplementary Material Zusatzmaterial
