# Supplementary material for: Differences in complications between colonoscopy and esophagogastroduodenoscopy in Japan using large-scale health insurance claims data
Source: Endosc Int Open. 2025 Sep 12;13:a26896049. doi: 10.1055/a-2689-6049 (PMC12445251; doi:10.1055/a-2689-6049)
Supplement: Supplementary file 1 — Supplementary Material [file 10-1055-a-2689-6049_26904312.pdf]

Supplementary Table 1 Japanese standard code for medical procedure about colonoscopy used in this study.

	Standard code for medical procedure	
CS without treatment	160094710	Colonoscopy up to sigmoid colon
	160094810	Colonoscopy up to descending colon
	160094910	Colonoscopy up to ascending colon or cecum
CS with lesions resection	150183410	Endoscopic mucosal resection of polyps ≥ 2 cm or others
	150183650	Endoscopic polypectomy of polyps < 2 cm in size or others
	150285010	Endoscopic mucosal resection to early malignant tumors or polyps < 2 cm in size
	150348510	Endoscopic mucosal resection to polyps ≥ 2 cm in size
	190181210	Endoscopic mucosal resection to polyps < 2 cm
	190181310	Endoscopic mucosal resection to polyps < 2 cm needing life restriction
	190181410	Endoscopic mucosal resection to polyps ≥ 2 cm
	190181510	Endoscopic mucosal resection to polyps ≥ 2 cm needing life restriction
	150363910	Endoscopic submucosal dissection
EGD without treatment	160093810	Esophagogastroduodenoscopy
EGD with lesions resection	150253510	Endoscopic mucosal resection for esophageal tumor
	150274810	Endoscopic mucosal resection for esophageal malignant tumor

Endosc Int Open | DOI 10.1055/a-2689-6049 | © The Author(s).

150336810	Endoscopic submucosal dissection for esophageal malignant tumor
150276410	Endoscopic submucosal dissection for early malignant tumor
150323010	Endoscopic submucosal dissection for early gastric malignant tumor
150417210	Endoscopic mucosal resection for early duodenal malignant tumor
150276310	Endoscopic mucosal resection for early malignant polyp
150164410	Endoscopic mucosal resection for other tumor

CS, colonoscopy; EGD, esophagogastroduodenoscopy.

Supplementary Table 2. Japanese standard disease code and medical procedures for hemorrhage used in this study

Complications	Standard Disease Code	
Hemorrhage	4489012	Hemorrhage from angioectasia
	8840294	Mallory-Weiss syndrome
	5308005	Esophageal hemorrhage
	5789001	Gastric hemorrhage
	5789007	Gastrointestinal hemorrhage
	5789008	Upper gastrointestinal hemorrhage
	9584004	Hemorrhagic shock
	8844477	Postoperative hemorrhagic shock
	9980002	Postoperative gastrointestinal hemorrhagic shock
	8842779	Hemorrhage due to biopsy
	5781002	Lower gastrointestinal hemorrhage
	Code for medical procedure	
	150263950	Endoscopic gastrointestinal hemostasis to the colonic bleeding
	150164850	Endoscopic gastrointestinal hemostasis to the rectal bleeding
	150224910	Blood transfusion (first time)
	150286310	Blood transfusion (≥2 times)

Supplementary Table 3 Japanese standard disease code for perforation used in this study.

Complications	Standard disease code	
Perforation	8845743	Sigmoidal perforation due to diverticulum
	5698003	Sigmoidal perforation
	8845750	Transversal colon perforation due to diverticulum
	8845764	Descending colon perforation due to diverticulum
	8842779	Rectal perforation due to trauma
	8845781	Colonic perforation due to diverticulum
	5698015	Colonic perforation
	8835346	Postoperative perforation
	8845807	Ascending colon perforation due to diverticulum
	8837748	Intestinal perforation
	8837749	Peritonitis due to intestinal perforation
	1541010	Perforation due to rectal cancer
	8847528	Perforation due to rectal diverticulum
	5694009	Rectal perforation
	8844903	Intestinal perforation during endoscopy
	8845817	Intestinal perforation due to diverticulum
	8845836	Cecal perforation due to diverticulum
	8839203	Intestinal perforation
	5694009	Rectal perforation
	5304001	Esophageal perforation
	8830483	Gastric perforation
	8835296	Duodenal perforation
	8845801	Duodenal-diverticula perforation
	8837748	Intestinal perforation

5679012	Peritonitis due to perforation
8836093	Abdominal abscess due to peritonitis
5678001	Duodenal-peroration peritonitis
5679006	Acute peritonitis
8844903	Endoscopic perforation

Supplementary Table 4 ACT code for antibiotics drugs used in this study.

Antithrombotic drugs	WHOACT code for drugs	
Antibiotics	J01C	Beta-lactam antibiotics, penicillin
	J01D	Other beta-lactam antibiotics
	J01M	Quinolone antibiotics
	J01R	Antibiotics combinations
	J01X	Other antibiotics

Supplementary Table 5 ICD code for medical procedure and drugs about fatal events used in this study.

Complications	ICD code for medical procedure	
Fatal events	J047	Electric counter shock
	J046	Heart massage
	J044	Endotracheal intubation
	J045	Artificial respiration
	K546	Percutaneous coronary angioplasty
	K547	Percutaneous coronary atherectomy
	K548	Percutaneous coronary angioplasty (specific catheter)
	K549	Percutaneous coronary stenting
	K550	Percutaneous coronary thrombus suction
	ICD code for drugs	
	C01CA03	Adrenalin
	C01CA04	Dopamine
	C01CA07	Dobutamine
	B01AD02	Alteplase
	N07XX14	Edarabone

Supplementary Table 6 WHOACT code for antithrombotic drugs used in this study.

Antithrombotic drugs	WHOACT code for drugs	
Anticoagulant	B01AA03	Warfarin
	B01AE01	Dabigatran
	B01AF01	Rivaroxaban
	B01AF02	Apixaban
	B01AF03	Edoxaban
Antiplatelet	B01AC06	Aspirin
	B01AC04	Clopidgrel
	B01AC22	Prasugrel
	B01AC24	Ticagrelor
	B01AC05	Ticlopidine
	B01AC23	Cilostazol
	B01AC19	Beraprost
	B01AC07	Dipyridamole
	B01AC	Ozagrel Sodium
	B01AC	Salpogrelate hydrochloride
	B01AC	Ethyl icosapentate
	B01AC	Limaprost alfadex
